# Determining geographic areas and populations with timely access to cardiac catheterization facilities for acute myocardial infarction care in Alberta, Canada

**DOI:** 10.1186/1476-072X-6-47

**Published:** 2007-10-16

**Authors:** Alka B Patel, Nigel M Waters, William A Ghali

**Affiliations:** 1 Department of Geography, University of Calgary, 2500 University Drive NW, Calgary, AB, T2N 1N4, Canada; 2 Faculty of Medicine, University of Calgary, 3330 Hospital Drive NW, Calgary, AB, T2N 4N1, Canada; 3 Department of Community Health Sciences, University of Calgary, 3330 Hospital Drive NW, Calgary, AB, T2N 4N1, Canada; 4 Centre for Health and Policy Studies, Faculty of Medicine, Department of Community Health Sciences, University of Calgary, 3330 Hospital Drive NW, Calgary, AB, T2N 4N1, Canada; 5 Department of Geography and Director, GIS Center of Excellence, George Mason University, 4400 University Drive, Fairfax, Virginia, 22030, USA

## Abstract

**Background:**

This study uses geographic information systems (GIS) as a tool to evaluate and visualize the general accessibility of areas within the province of Alberta (Canada) to cardiac catheterization facilities. Current American and European guidelines suggest performing catheterization within 90 minutes of the first medical contact. For this reason, this study evaluates the populated places that are within a 90 minute transfer time to a city with a catheterization facility. The three modes of transport considered in this study are ground ambulance, rotary wing air ambulance and fixed wing air ambulance.

**Methods:**

Reference data from the Alberta Chart of Call were interpolated into continuous travel time surfaces. These continuous surfaces allowed for the delineation of isochrones: lines that connect areas of equal time. Using Dissemination Area (DA) centroids to represent the adult population, the population numbers were extracted from the isochrones using Statistics Canada census data.

**Results:**

By extracting the adult population from within isochrones for each emergency transport mode analyzed, it was found that roughly 70% of the adult population of Alberta had access within 90 minutes to catheterization facilities by ground, roughly 66% of the adult population had access by rotary wing air ambulance and that no population had access within 90 minutes using the fixed wing air ambulance. An overall understanding of the nature of air vs. ground emergency travel was also uncovered; zones were revealed where the use of one mode would be faster than the others for reaching a facility.

**Conclusion:**

Catheter intervention for acute myocardial infarction is a time sensitive procedure. This study revealed that although a relatively small area of the province had access within the 90 minute time constraint, this area represented a large proportion of the population. Within Alberta, fixed wing air ambulance is not an effective means of transporting patients to a catheterization facility within the 90 minute time frame, though it becomes advantageous as a means of transportation for larger distances when there is less urgency.

## Background

### Cardiac catheterization and the importance of the 90 minute transfer time

Myocardial infarction (MI) arises from the blockage of blood flow to the heart and is commonly known as a heart attack. The U.S. National Library of Medicine and the National Institutes of Health have compiled an on-line encyclopedia that highlights the treatment options for MI. The treatments can be summarized under four headings: pain control medications, blood thinning medications, other medications, and surgery and other procedures.

There have been several studies in recent years that have shown the superiority of procedures such as catheterization and early angioplasty (with or without stenting) as treatment for acute myocardial infarction (AMI). When the AMI type is an ST segment elevation myocardial infarction (STEMI – a subtype of AMI diagnosed by an electrocardiogram), immediate re-establishment of the blood flow is desirable. Early catheterization has been directly compared to the use of blood thinning medications such as thrombolytic therapy, and shown to reduce mortality rate and recurrence of myocardial infarction [1-4]. However, the success of using catheterization to treat STEMI is dependent on a number of factors; one of the most documented influences on its success is the time to treatment.

Early catheterization permits the rapid detection and localization of arterial blockages that can then be treated with percutaneous coronary intervention (PCI). This is a procedure involving catheter passage through the blocked area, balloon inflation to dilate the affected artery, and in most cases, insertion of a metal stent that helps to keep the artery open. Delaying PCI ultimately results in an increase in the mortality rate for patients [5]. Current American and European guidelines suggest performing cardiac catheterization within 90 minutes of the first medical contact [6,7]. For this reason, this study focuses on determining the populated places that are within a 90 minute transfer time to a city with a catheterization facility. This determination is crucial as the cardiac catheterization procedure is time sensitive [8].

The time sensitive nature of catheter intervention for AMI necessitates that access to cardiac catheterization facilities be studied. Within the province of Alberta (Canada), there are three cardiac catheterization facilities. One is located in Calgary at the Foothills hospital, and two are located in Edmonton at the Royal Alexandra hospital and the University of Alberta hospital. Because access to these facilities is dependant in part to the geographic location of patients, geographic information systems (GIS) are a potentially valuable tool for studying this problem.

### GIS in accessibility analysis

Accessibility measures are commonly integrated into GIS studies regarding access to health services. There are a number of studies in recent years that have utilized GIS as a tool for evaluating accessibility to health services [9-12]. In addition to evaluating accessibility to health services, GIS has proven to be an effective tool for evaluating emergency transport decisions to these services. One study considered how GIS could be used as a tool to help make transport decisions for trauma patients [13]. This study is of interest because its goal was to create a map that would show where air or ground ambulance would be preferred to transport patients to a trauma centre, in terms of faster travel time.

In this paper, we use GIS and reference travel time data from the regional air ambulance provider to: 1) Determine the areas within the province where transfer to a hospital for catheter intervention would be the preferred method of treatment for AMI and evaluate the proportion of the adult population living within these defined areas, and thus having rapid access to catheter intervention for AMI; and 2) Determine which areas of the province are best served by a certain mode of transport in terms of fastest travel time.

## Methods

### Study area

**Figure 1:**
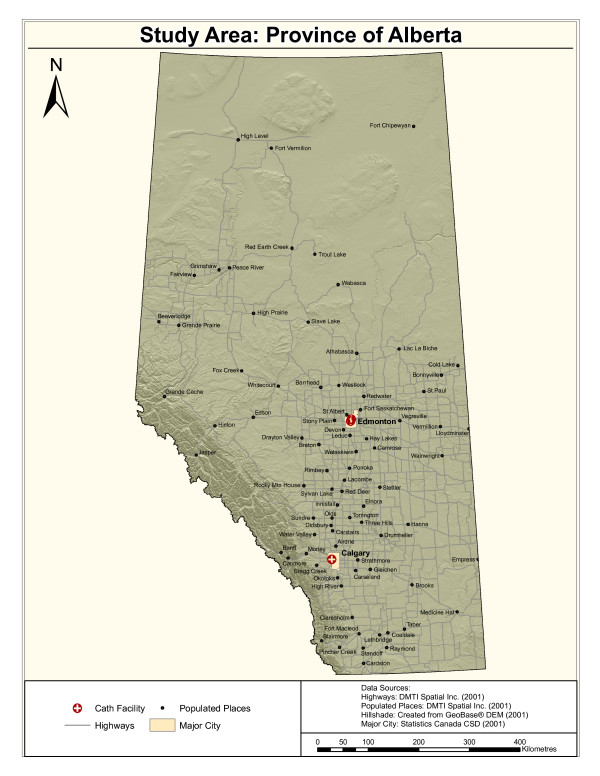
Study Area: Province of Alberta.

The area of focus for this study is the province of Alberta, a western Canadian province with a population of approximately three million. Figure 1 shows the extent of the study area; highlighted are the three catheterization facilities that are of interest for this project. For reference purposes, this figure includes the populated places with a population greater than 2000 within the provincial boundaries. The populated places are the municipalities that are typically the originating points for patient transfer to the larger cities with catheterization facilities. There are 136 different municipalities that are included in this study and two major cities within the province where the catheterization facilities are located. One catheterization facility is located in the city of Calgary and two are located in the city of Edmonton. For the purpose of this study the two catheterization facilities in Edmonton are treated as one due to their close proximity and the small scale of this analysis.

Figure 1 also includes the road networks that are designated as 'highways'. The southern area of the province contains a much denser network of highways than the northern area. This is an important consideration when patients are being transported by ground. A subset of the road networks are included in this figure because in this study, the distance travelled by ground ambulance is calculated along the road network. One final interesting feature of the province that can be ascertained from the map in Figure 1 is the terrain. It can be seen that along the south-western boundary of the province (which is adjacent to the province of British Columbia) there is a range of mountains (Rocky Mountains). In this area the road networks and populated places are sparse. Although the terrain data shown in the figure are not explicitly used for analytical purposes, these data provide an impression of the physical environment within the province.

### Data Source: Alberta Chart of Call

To study the accessibility of areas in terms of travel time, it was necessary to obtain data on travel times from different originating locations within the province to the catheterization facilities within Calgary and Edmonton. Although it was not possible to obtain detailed individual trip records within the time constraints of this study, it was possible to obtain generalized reference data on travel times via different modes of transportation. An integral data set used in this study is the Alberta Chart of Call. The Chart of Call is used in the Alberta Shock Trauma Air Rescue Society's (STARS) Emergency Link Centre and its primary objective is to "assist dispatchers in choosing the most appropriate air carrier and medical crew once a medical decision has been made that a patient needs to be transported" [14]. The data within this manual are collected and compiled by the Provincial Flight Coordination Centre and STARS. The three different modes of transportation accounted for in this reference manual are rotary wing air ambulance (RW), fixed wing air ambulance (FW) and ground ambulance (GRD).

Table 1 shows an example of the data used from the Chart of Call for the municipality of Drumheller, Alberta. For each municipality there is one record that contains different options for patient transport to a city with a tertiary care centre. Within the Chart of Call, the overall travel times to tertiary care for the two air ambulance options (FW and RW) are calculated based on average yearly response times; the individual records used to obtain the averaged time were not available for this study. The ground ambulance times are calculated using a fixed calculation of the distance along the road network from city centre to city centre divided by 100 km/hr with the addition of 10 minutes for in city travel added at both the origin and destination. Although this fixed travel time may seem unrealistic for road travel, the consideration must be made that ambulances often travel at speeds above the posted speed limit; the fixed addition of 10 minutes at both the origin and destination accounts for the impedance encountered through in-city travel. In the absence of individual travel time records to evaluate the air and road travel time and variability encountered with varying conditions, the Chart of Call data was deemed a credible data source in this study as the time calculations were based on consultation with the stakeholders of the Alberta Air Ambulance Program [14].

**Table 1:** Example of travel time data from Chart of Call for Drumheller, AB

**Origin and Mode**	Time to Patient*	Out of Hospital Time*	Time to Tertiary Care*
Drumheller GRD**	0	103	103
Calgary RW**	44	38	112
Calgary FW**	58	60	148
Medicine Hat FW**	92	60	182

* Time to Patient is the time from dispatch until the response team arrives at the sending hospital. *Out of Hospital Time *is the time that the patient is out of a hospital while being transported from the sending facility to the tertiary care centre. *Time to Tertiary Care *is the total time from dispatch to arrival at the tertiary care centre. Note: Both air modes contain a fixed addition of time in the final *Time to Tertiary Care *calculation called *'30-minute in hospital time'*, which accounts for patient stabilization and preparation for transport [14].

** (Abbreviations: GRD = ground ambulance; RW = rotary wing air ambulance; FW = fixed wing air ambulance)

Included in the final time to tertiary care are fixed loading and unloading times associated with all three modes. Both air modes also contain a fixed addition of time in the calculation called '30-minute in hospital time', which accounts for patient stabilization and preparation for transport; there is no such time assigned to ground transport in the Chart of Call because there is typically less time involved in preparing a patient for transport via ground. Air ambulance time calculations also contain a 'time to patient' that represents the time from dispatch to the time the air carrier reaches a patient. Ground ambulances are more typically ready to transport a patient and thus in the Chart of Call calculations there is no addition of 'time to patient' for the ground mode of transport. The application of this generalized data set to this study is justified by its daily use as part of the decision making process for patient transport within the province of Alberta.

### Interpolating travel time surface

**Figure 2:**
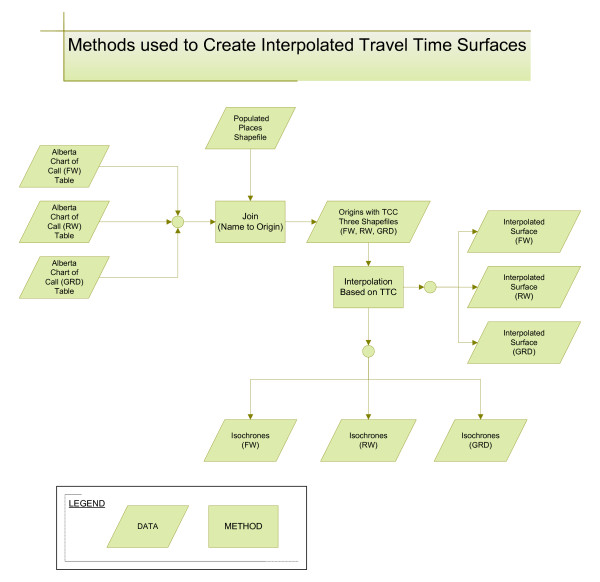
**Methods used to create interpolated travel time surfaces**. (Abbreviations: GRD = ground ambulance; RW = rotary wing air ambulance; FW = fixed wing air ambulance; TTC = time to tertiary care)

**Figure 3:**
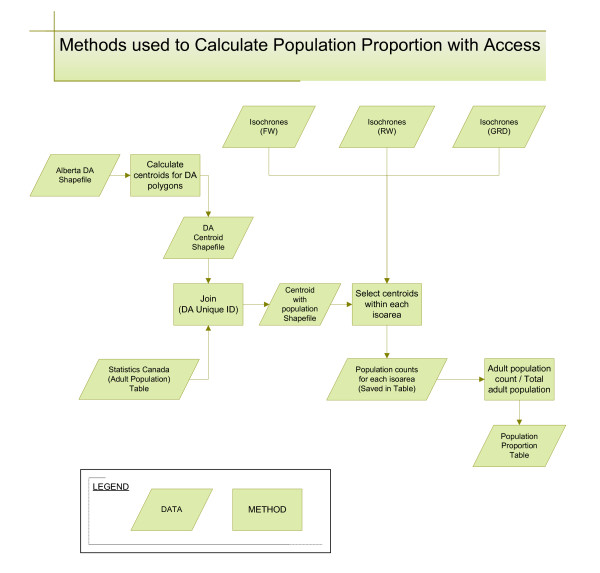
**Methods used to calculate population proportion with access**. (Abbreviations: GRD = ground ambulance; RW = rotary wing air ambulance; FW = fixed wing air ambulance; DA = dissemination area)

In this study the method of spatial interpolation was used to create a continuous surface of patient transport times for the different modes of transport. Given a set of point locations with known values over a surface, the method of spatial interpolation finds a "function that will best represent the whole surface and that will predict values at other points or for other sub areas" [15]. The Chart of Call records (an example is shown in Table 1) were compiled into three separate tables, each representing one mode of transport. The individual tabular files for the three modes of transport were ultimately converted into a continuous surface of travel times within ESRI ArcGIS 9.1 software [16]. Figures 2 and 3 highlight the data, methods and general processes used in this study.

First, each of the Chart of Call modes was individually joined to the Populated Places point file. This Populated Places file was created by DMTI Spatial [17] and represents the municipalities or originating points of travel to a city with a catheterization facility. Although there were 136 municipalities used in this study, each was not always serviced by all three modes. Joining the Chart of Call records to point locations within a GIS made it possible to give each origin's time to tertiary care a geographic location. In this study only the fastest travel time was used. For example, if there was an originating location in the Chart of Call that had multiple options for FW transport, only the fastest FW transport time was joined to the populated places point. For example, in Table 1 there are two options for FW transport from Drumheller to Calgary. One option is a FW originating in Calgary and the other is a FW originating in Medicine Hat. Since the Calgary FW has a faster travel time than the one originating in Medicine Hat, only the Calgary FW data is included in this study.

Once these joins were made to shortest travel times, there were a total of 99 FW points, 109 RW points and 126 GRD points available for the subsequent interpolation. A variety of interpolation methods were investigated during the preliminary stages of analysis (e.g. inverse distance weighting, radial basis function, global and local polynomial methods and kriging). Local polynomial interpolation was selected because this method had the best fit to the data available for each transportation mode. The travel time data used for this study were averaged over different seasons and times of day and represented a subset of the municipalities within the study area; consequently there was a need to match the interpolation method to the data used.

Inverse distance weighting and radial basis function (both exact methods of interpolation that honor the original data points) created surfaces that were irregular with the appearance of 'bull's eyes' in some areas due to the distribution of the point data. Global polynomial interpolation created an overly generalized surface while kriging produced a surface with a false sense of precision. Since the data were averaged, a method was needed that would show the changes in the travel times over the study area without creating a false sense of precision in the results. Local polynomial interpolation was the best method for this. There are a variety resources available to researchers which discuss in detail the theoretical basis of the different interpolation methods [15,18-20].

Figure 2 reveals that the method of interpolation created three surfaces and isochrones: a set of interpolated surfaces and isochrones for each of the modes of transport (FW, RW, and GRD). The three interpolated surfaces were converted to raster data files using ArcGIS 9.1. Raster data files were created in order to compare the study area for the three modes on a cell by cell basis to determine the mode with the fastest travel time for each cell. Using the *Raster Calculator *tool in ArcGIS, the three modes were compared using conditional statements. The following is an example of the statement used to find the areas with the fastest travel times for GRD: GRDfaster = con([GRD] < [FW], con([GRD] < [RW], 3, 2), 1). Using this statement, the cells where GRD transport had the lowest travel time in comparison to the other modes, were given a value of 3.

In order to create a visualization comparing all three modes of transport, three surfaces were created that showed areas where each mode was the fastest means of patient transport (e.g. one surface showing where RW was fastest, one where FW was fastest and one where GRD was fastest). Once the fastest areas for all three modes were created, it was possible to overlay these three layers onto one another to create a visualization of the locations where each mode of transport would be preferred for patient transport to a city with a tertiary care centre.

### Evaluating the population with access

Once the interpolated surfaces were created, the results were displayed as isochrones and these isochrones were then converted to a shapefile, which is a vector data storage format used within ESRI software. An isochrone is a linear representation of an equal time interval from a specified location and thus can be used to display results from an accessibility analysis in terms of travel time. The creation of the isochrone lines made it possible to begin extracting the adult population (20 years of age and older) having access within different travel time intervals. The census unit used for this study was the Statistics Canada dissemination area. Dissemination areas (DA) are the smallest geographic area for which census data are disseminated. Each DA contains roughly 400 to 700 people and respects the boundaries of the census tracts and census subdivision so they remain relatively stable over time [21].

Figure 3 highlights the data and methods used to evaluate the population proportion with access. First, using the DA polygon shapefile, calculations were made within ArcGIS to find the centre of the DA boundaries. These locations, called centroids, were represented by a point shapefile and used to join to the population data that was in tabular format. The isochrone layers were then overlaid onto the DA centroids and a *select by location *operation was performed within ArcGIS to extract the population (in the form of DA centroids) within different isoareas. Those centroids were selected which *have their centre in *a specific isoarea; this data set contained no centroids that fell directly on an isochrone line. Using a method that selected points from areas ensured that the population was not double counted. The misclassification of DA centroids (a DA being included or excluded from within an isochrone) could occur, but the error created would be random and non-directional. These methods were developed based on processes used in previous studies [10,11].

The population counts within each time interval were transferred to a tabular file and then converted to proportions of total population. This would allow for an estimate of the population proportion with access within selected travel time minutes. This study used the most current available data which were collected in the 2001 census when the total population of Alberta was calculated to be 2977625 people. Details on the collection of the Statistics Canada census data can be found in the 2001 Census Handbook [22].

## Results

### Areas with access to catheterization facilities

**Figure 4:**
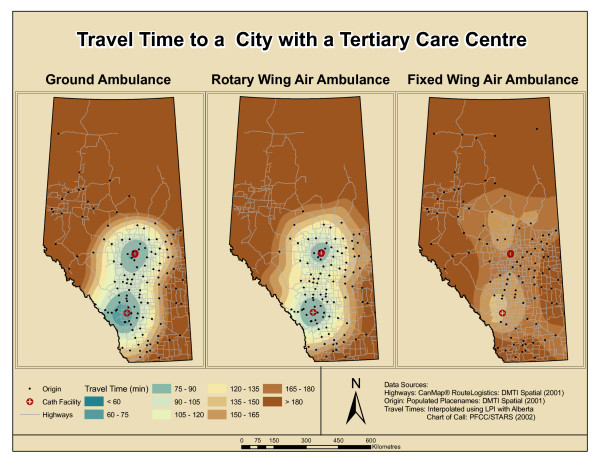
**Travel time to city with a tertiary care centre**. (Abbreviations: LPI = local polynomial interpolation)

Figure 4 shows the time to tertiary care for the three modes of transportation presented side by side for comparative purposes. When viewing the map for ground ambulance transport, the origin points used for the interpolation are noticeably concentrated in the southern and western areas of the province. For the rotary wing ambulance, the points are concentrated only in the southern areas of the province within 250 km of the centres of Calgary and Edmonton (where the catheterization facilities are located). For the fixed wing air ambulance, the points used for the interpolation are spread more evenly throughout the province but the concentration of points is in the central eastern side of the province. Additionally, for the fixed wing mode there are fewer points within 100 km of the catheterization facilities.

The travel times interpolated using these points show visible trends. The ground ambulance travel times within 90 minutes of a city with a tertiary care centre cover a larger area than the area covered by the rotary wing air ambulance. When comparing GRD to RW travel times between 120 and 180 minutes, there is a larger distance covered in the same amount of time by RW. The larger distance covered by the RW mode is represented through the wider time intervals in the RW interpolation.

Figure 4 shows that fixed wing travel times are higher around the major cities in comparison to GRD or RW, but this mode has lower times for areas that are further north than the other two modes of transport. There is a clear asymmetry in the appearance of travel times via fixed wing air ambulance. While the RW and GRD times appear to increase radially from the two major cities, the fixed wing travel times do not exhibit this same pattern.

### Population with access

Table 2 shows the proportion of the total adult population within the three major isoareas of interest (90, 120 and 180 minutes). The table shows that roughly 70% of Alberta's adult population has access within 90 minutes by ground and roughly 66% has access within the same time interval by rotary wing. This equates to 74370 more people having access within 90 minutes by ground than by air. Within 120 minutes, roughly 80% have access by ground ambulance and roughly 78% have access by rotary wing. From the table, it is seen that there is no population that has access within 120 minutes using the fixed wing mode of transport.

**Table 2:** Population proportion within each isoarea for GRD, RW and FW

**Time to Tertiary Care**	Within 90 minutes	Within 120 minutes	Within 180 minutes
**Ground**	0.696	0.801	0.894
**Rotary Wing**	0.661	0.782	0.909
**Fixed Wing**	0	0	0.872

The percentage of the population within 120 minutes is only slightly higher using ground over rotary wing air ambulance. There are 40865 more people who have access by ground at 120 minutes when compared with rotary wing for the same time interval. For 180 minutes the proportion of population becomes higher for RW than for GRD. Also, within 180 minutes transport time to a tertiary care facility, FW becomes an option for patient transport from some regions. Using the Chart of Call data it was found that the proportion of population for fixed wing transport is lower for this time interval than for both RW and GRD. At 180 minutes there are 30715 more people with access by RW over GRD and 79220 more people who have access by RW over FW.

### Comparison of different modes

**Figure 5:**
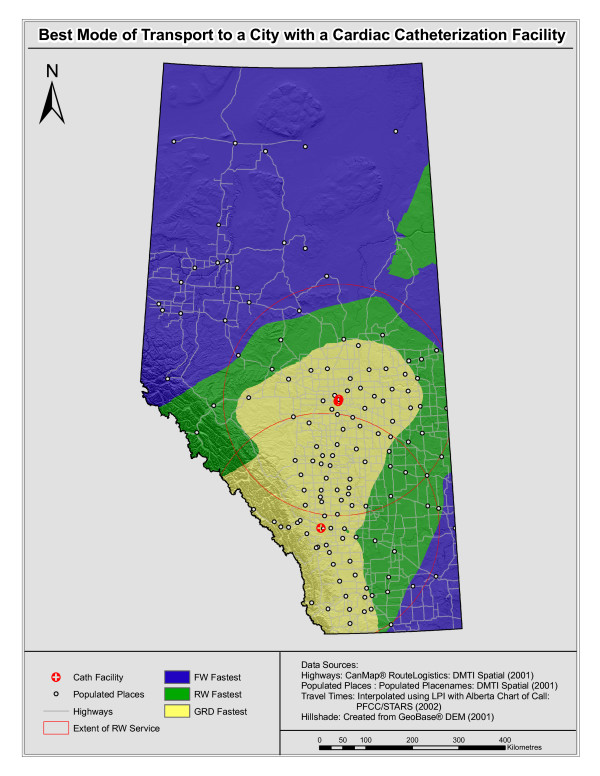
**Comparison of three modes of transport**. (Abbreviations: GRD = ground ambulance; RW = rotary wing air ambulance; FW = fixed wing air ambulance; LPI = local polynomial interpolation)

Figure 5 is a visualization showing where each mode would be faster throughout Alberta. In this figure areas where FW would be the fastest mode for transporting a patient to a tertiary care centre are shown in blue, those where RW would be fastest are shown in green and those where ground would be fastest are shown in yellow. The red circles represent the 250 kilometre extent of RW air ambulance service. The limitation of distance flown by the current helicopters is due to the maximum distance this mode can fly without refuelling.

The areas closer to the major cities, where the road network is denser, are the areas where using ground ambulance for patient transport would be preferred. Within the RW service area, it appears that benefit is greater as one moves further from the city. The areas within the rotary wing ambulance service extents are best served by rotary wing when comparing RW to FW. There is a very small area to the east of Calgary where it appears that RW is faster than GRD. Finally, there is an area in the north-eastern portion of the province where travel is deemed to be faster by RW than by FW.

## Discussion

### Areas and population with access to catheterization facilities

Our findings show that only rotary wing and ground ambulance are viable options for patient transport to catheterization facilities within 90 minutes. It was found that roughly 70% of Alberta's adult population had access to a city with a catheterization facility by ground transport within the 90 minute constraint while roughly 66% of the adult population had access by rotary wing air transport. This is lower than the national level of access in the United States where it was found that by ground, nearly 80% of the adult population lived within 60 minutes of a hospital with a cardiac catheterization facility [23]. The lower population density and typically lower number of facilities within the province of Alberta when compared with the US as a whole is one explanation for the lower population proportion with geographic access.

The level of access in Alberta is also lower than the population access to tertiary care facilities in Wales, UK, where one study estimated that between 73–78% of the population had access within 60 minutes by ground and between 84–91% had access within 90 minutes by ground to tertiary care facilities [10]. There were five cities with tertiary facilities in Wales and the surrounding area of the UK that were included in this study. The higher numbers of facilities, higher population density and shorter distance to travel to the nearest facility are probable explanations for the differences seen between Wales and Alberta. In our study and in the studies discussed above, the primary focus was on geographic access. Individual access, which would be affected by socio-economic conditions and other factors, was not directly assessed in these studies.

The expectation may be to see an increase in the population proportion with access when a patient is being transported by helicopter, but there are initial time costs involved with patient transport by air (e.g. time to patient, time for patient stabilization and preparation for transport). These initial time costs result in ground being a better option for transport until the threshold of the fixed time costs are overcome. Other studies have also found that areas closest to the destination facilities are best served by ground transport due to the distance from the helicopter base to the scene and due to the time necessary to deploy such vehicles [13,24]. The areas where helicopter is faster than ground transport can be thought of as areas where this initial time cost has been overcome. Based on the population study, it appears that this cost is overcome between 120 and 180 minutes; this is seen in the higher proportion having access within 180 minutes by helicopter than by ground.

Figure 4 showed that the areas accessible within 90 minutes by helicopter were concentric around the major cities. This is a result of the flight pattern for air transport that would be similar to a straight line distance, thereby making the travel times and distances correlated [25]. The rotary wing travel times outside of a 250 kilometre radius of Calgary and Edmonton can be disregarded since the current rotary air ambulance service in Alberta only flies within these limits. Although the fixed wing air ambulance proved to be unsuitable for transporting AMI patients, the results revealed that this mode of transport could reduce the patient transport time for areas that are outside the RW service area. This is an important finding when considering patients who do not need to be transported within 90 minutes to a city with a tertiary care centre (i.e. for less urgent tertiary services).

The asymmetry for FW travel time around the city centres is likely due to the variation in the start location for this mode. When fixed wing is destined for Calgary the origin of the FW is Calgary in the majority of cases (i.e. a round trip has to be made). In contrast, many fixed wing ambulances which are destined for Edmonton are dispatched from centres such as Lac La Biche, Grande Prairie, Fort McMurray, Peace River and Slave Lake (See Figure 1). This difference in origins for the FW ambulances causes the asymmetry in travel times around Edmonton for this mode. The variations in the results between GRD, RW and FW transport seen in Figure 4 show that the methods used in this study can help to gain a visual understanding of those areas within the province where transport to a catheterization facility should be the preferred method of treatment.

### Comparison of different modes

In addition to the findings specific to AMI care, some general findings on the nature of GRD, RW and FW transport within Alberta were also uncovered. It was found that closer to the cities with tertiary care facilities, GRD would be the fastest mode of transport. Beyond a 100 km radius of the two major cities, RW started to become a faster mode of transporting patients; this was especially apparent in the eastern and northern parts of the province within the RW service area.

Although the three mode comparison provided some generalizations on travel times for the different modes of transportation, it is believed that the comparison between GRD and RW does not show the true delineations of the areas where transport would be faster by air. The Alberta Chart of Call data used in this study were for populated areas, most with a community hospital. There are a number of rural areas along the Alberta-British Columbia provincial borders that are not easily accessible by road. Areas without access to a major highway would be areas where it is expected that patient transport would be faster using RW over GRD. The generalization using interpolation mutes these finer delineations between areas where one mode would be preferred over another due to the sparseness of data in this area. Previous studies that determined the appropriate mode of patient transport within different areas found that these finer delineations were possible when actual trip records were used [13]. Obtaining individual trip records for GRD as well as a travel times for RW from locations that are not populated would provide a better indication of where different modes would be preferred for patient transport.

Outside of the RW service areas, fixed wing ambulance would always be the preferred mode of transporting patients to a tertiary care centre in Calgary or Edmonton. The results of the comparison between FW and RW showed that RW would be preferred along the north-eastern edge of the province. This result is due to the sparseness of points used in the interpolation for northern areas and can be disregarded since the current rotary wing air ambulance does not service these areas. The very small area east of Calgary where RW is faster than GRD has travel times that are less than a minute faster by RW. The area surrounding the RW patch is in an area where the travel time values for RW and GRD are similar; the triangular distribution of the origin points and the comparable values for RW and GRD travel times interpolated in this area are most likely the reason for the appearance of this small patch of preferred RW. As future RW ambulances have the potential to increase both speed and range, there is potential for our findings to be reconsidered in step with new technology.

Prior studies have compared accessibility to hospitals in terms of travel time for rotary wing and ground ambulance transport [13,24,26]. There have also been specific studies performed to understand national level access to cardiac catheterization facilities in terms of ground ambulance travel times [23]. This study contributes to current literature on access to tertiary care facilities by comparing three modes of emergency transport. The data used for the study conducted in this paper allowed for a regional understanding of the accessibility of cardiac catheterization facilities to the general population using ground, helicopter and fixed wing emergency transport. Determining the areas with access within 90 minutes to a cardiac catheterization facility gives an indication of where direct referral to these facilities would be preferred over transport to a non-catheterization facility.

### Caveats and future research

The time constraint used in this study is based on current guidelines but is not inflexible. The limit of 90 minutes is somewhat arbitrary and clinical judgement is ultimately needed in order to determine if patient transport to a catheterization facility is the best option for treatment even if the 90 minute time period is exceeded. The fact that all AMI do not have the same risk is another consideration that must be made when making transport decisions. Another aspect of time important to consider is the *door-to-balloon *time, which is the time from patient presentation to the hospital to reperfusion. The study in this paper considers the patient transport time from dispatch to the door of the hospital. The delays encountered within the hospital are not considered.

We remind readers that in discussing AMI in this study, our specific condition of focus is STEMI (ST segment elevation myocardial infarction). This condition can be distinguished from other acute coronary syndromes through the use of an electrocardiogram (ECG). One method which could be incorporated into the patient transport process to reduce the *door-to-balloon *time is a STEMI program where ambulance attendants are trained to diagnose STEMI from the ECG; once a STEMI is diagnosed en route the patient can be transported directly to a catheterization facility, thus eliminating the need for transfer from a facility which does not provide catheterization services [27]. Furthermore, such care pathways can incorporate expedited transfer through emergency room bypass to reduce door-to-balloon times (i.e. patients are sent directly to the catheterization lab).

Although some areas may show that ground is the faster means of transporting a patient, there are various considerations that must be made when choosing the mode of patient transport. Through personal communications with the STARS emergency link centre staff it was uncovered that the final decision for patient transport at the link centre was made by an Emergency Referral Physician (ERP). Some of the factors that affect transport mode decisions relate to the patient's condition, time for patient preparation and level of in-transport care needed.

When patients are transported by air there is the possibility for an in-flight physician to be present thereby allowing patient access to medical care before they reach the hospital. One study found that cardiac patients transported by air presented to hospitals with reduced chest pain, a result of the medical care given en route [26]. It is therefore important to consider that the fastest mode may not always be the 'best' mode in certain conditions. Issues such as time to patient or availability of RW influence the decision of using RW, while time of day and congestion can dissuade the use of GRD. While geography can aid in this decision making process, the ultimate decision is a clinical one based on patient needs and their requirements for transport.

The methods and data used for this study proved to be effective for generalizations but the reader should be aware that different interpolation methods yield dissimilar surfaces. Also, interpolation methods are sensitive to the sample size (number of points used) and the density or sparseness of the points. For the purpose of this study it was decided that only the general trend of the travel times was desired. Using interpolation methods that would create highly irregular time surfaces gave a sense that variation in the travel times were being uncovered that were not possible with the use of these generalized data. Increasing the number and distribution of originating points of travel would allow for the use of alternative interpolation methods and ultimately increase the precision of this study. One study showed that using a large database of originating points for patient travel time allowed for the delineation of confidence intervals around travel time zones created using the method of interpolation [13].

Obtaining data in the form of individual trip records would allow for the finer details of accessibility via the different modes to be uncovered. Individual records would also be valuable in determining the variability in response times with highway density, terrain conditions, time of the day and weather conditions. A future research goal is to obtain individual travel time records for the different modes to uncover the variability in travel times over these different conditions. Using finer data and exact interpolation methods would allow for the creation of a surface that stayed true to the values at the original data set locations. Additionally, using individual data would allow for sensitivity testing across the different spatial interpolation methods to quantify the differences produced in the results.

There are other important research directions which can be taken using this study as a basis. Since the census data used in this study were collected in 2001, there will be a need to reevaluate the results using 2006 census data when the new census data become publicly available. Within this five year period, there has been an increase in the population in the northern areas of Alberta. Since this is an area that is not currently accessible within the recommended time frame, future studies using GIS could evaluate how the addition of a facility in northern Alberta would affect the population proportions with access. The addition of such a facility would of course be dependent on both the financial and staff resources available in this area.

## Conclusion

The results of this analysis have shown that the areas within the province that are accessible to catheterization facilities within 90 minutes are limited. Although these areas with access are limited, there is a high percentage of the total population within these regions, due to the concentration of the population within and surrounding the major city centres of Calgary and Edmonton. Ground and rotary wing ambulance are the two modes of transportation that make access to a catheterization facility within the 90 minute target time frame possible. Direct transfer of AMI patients to the nearest cardiac catheterization facility within these areas would be preferred based on current outcomes research.

This paper has shown that GIS is a powerful tool that can be used to determine accessibility to catheterization facilities. However, data limitations dictate that the results are generalizations and should be considered with some caution. In order to make more accurate determinations of areas with access to catheterization facilities it is necessary to obtain individual trip records of patient transport via the different modes.

## Competing interests

The author(s) declare that they have no competing interests.

## Authors' contributions

ABP compiled data, performed analysis and drafted the manuscript. NMW participated in the design of the study, consulted on the geographic techniques to be used and provided comments on the manuscript. WAG conceived the study, and participated in its design and coordination and provided comments on the manuscript. All authors read and approved the final manuscript.
